# Maternal cell phone use in early pregnancy and child’s language, communication and motor skills at 3 and 5 years: the Norwegian mother and child cohort study (MoBa)

**DOI:** 10.1186/s12889-017-4672-2

**Published:** 2017-09-05

**Authors:** Eleni Papadopoulou, Margaretha Haugen, Synnve Schjølberg, Per Magnus, Gunnar Brunborg, Martine Vrijheid, Jan Alexander

**Affiliations:** 1 Department of Environmental Exposures and Epidemiology, Division of Infection Control and Environmental Health, Norwegian Institute of Public Health, PO Box 4404, 0403 Oslo, Norway; 2 Department of Environmental Exposures and Epidemiology, Division of Infection Control and Environmental Health, Norwegian Institute of Public Health, PO Box 4404, 0403 Oslo, Norway; 3 Department of Child Development, Division of Mental Health, Norwegian Institute of Public Health, PO Box 4404, 0403 Oslo, Norway; 4 Division of Health Data and Digitalisation, Norwegian Institute of Public Health, PO Box 4404, 0403 Oslo, Norway; 5 Department of Molecular Biology, Division of Infection Control and Environmental Health, Norwegian Institute of Public Health, PO Box 4404, 0403 Oslo, Norway; 6 ISGlobal- Barcelona Institute for Global Health, Doctor Aiguader, 08003 Barcelona, Spain; 7 Pompeu Fabra University, Barcelona, Spain; 8 Spanish Consortium for Research on Epidemiology and Public Health (CIBERESP), Instituto de Salud Carlos III, Madrid, Spain; 9 Division of Health Data and Digitalisation, Norwegian Institute of Public Health, P.O. Box 4404, NO-0403 Oslo, Nydalen Norway

## Abstract

**Background:**

Cell phone use during pregnancy is a public health concern. We investigated the association between maternal cell phone use in pregnancy and child’s language, communication and motor skills at 3 and 5 years.

**Methods:**

This prospective study includes 45,389 mother-child pairs, participants of the MoBa, recruited at mid-pregnancy from 1999 to 2008. Maternal frequency of cell phone use in early pregnancy and child language, communication and motor skills at 3 and 5 years, were assessed by questionnaires. Logistic regression was used to estimate the associations.

**Results:**

No cell phone use in early pregnancy was reported by 9.8% of women, while 39%, 46.9% and 4.3% of the women were categorized as low, medium and high cell phone users. Children of cell phone user mothers had 17% (OR = 0.83, 95% CI: 0.77, 0.89) lower adjusted risk of having low sentence complexity at 3 years, compared to children of non-users. The risk was 13%, 22% and 29% lower by low, medium and high maternal cell phone use. Additionally, children of cell phone users had lower risk of low motor skills score at 3 years, compared to children of non-users, but this association was not found at 5 years. We found no association between maternal cell phone use and low communication skills.

**Conclusions:**

We reported a decreased risk of low language and motor skills at three years in relation to prenatal cell phone use, which might be explained by enhanced maternal-child interaction among cell phone users. No evidence of adverse neurodevelopmental effects of prenatal cell phone use was reported.

**Electronic supplementary material:**

The online version of this article (doi:10.1186/s12889-017-4672-2) contains supplementary material, which is available to authorized users.

## Background

The use of equipment that emits radio frequency electromagnetic field (RF-EMF) has increased tremendously during the last 30 years and human exposure is widespread. The most frequently used technology relates to cell phones. In 2012, a Norwegian Experts Committee, established by the Norwegian Ministry of Health and Care Services, reviewed the evidence on possible negative health effects from weak RF fields [1]. After assesing a large number of studies, they concluded that no evidence of adverse health effects from exposure to weak RF fields was found. Other expert reviews, intitated also by the increasing public concern, have reported similar conclusions [2–4]. However there were few studies on reproductive and offspring’s developmental health, including neurodevelopment.

In a series of studies within the Danish National Birth Cohort, associations between maternal and child cell phone use and developmental milestones and behaviour of children were investigated [5–7]. Information on maternal cell phone use was collected retrospectively when the child was 7 years old. These studies reported an increased odds ratio for problematic behaviour at 7 years of age related to cell phone use during pregnancy [5, 6]. However, no association was found between cell phone use during pregnancy and offspring’s developmental milestones at 6 and 18 months of age [7]. In a Dutch birth cohort study, no association was found between prenatal exposure to cell phones or cordless phones and behavioural problems at the age of 5 years [8]. In a Spanish birth cohort study researchers found no associattions between maternal cell phone use during pregnacy and child’s early mental development [9]. However this is the only study in which child’s mental and psychomotoric development was assesed by phychologists, while the number of partcipants was lower than the Dutch and Danish studies.

As cell phone use has become abundant, cell phone based interventions and monitoring are applied also in the field of maternity and antenatal health care because of it’s low-cost. It has been shown to be also a relatively effective tool of public health promotion, especially in developing countries [10, 11]. However, the other side of the coin is that is important to investigate if there are any health effects related to exposure to electromagnetic fields during critical developmental periods, such as the intrauterine life and early childhood.

The aim of this study was to investigate any association between maternal phone use during first trimester and a) language skills at 3 years, and b) communication, gross and fine motor skills at 3 an 5 years of age, in a large prospective birth cohort.

## Methods

### Study population

Our study is conducted within the MoBa, which is a prospective population-based pregnancy cohort study conducted by the Norwegian Institute of Public Health [12]. Pregnant women from all over Norway were recruited from 1999 to 2008 at 17–18 weeks of pregnancy and 40.6% of invited women consented to participate. There are 114,500 children, 95,200 mothers and 75,200 fathers recruited in the cohort. Data used in this study are based on version 8 of the quality-assured data files, released for research in February 2014. The establishment and data collection in MoBa has obtained a licence from the Norwegian Data Inspectorate and approval from The Regional Committee for Medical Research Ethics. This study was approved by the Regional Committee for Medical Research Ethics in South-Eastern Norway

There were 96,875 singleton, live born pregnancies with no malformations and chromosomal anomalies. After excluding women with missing information in cell phone use in the first trimester (*n* = 9843), as well as in parity, maternal age, maternal education, year of delivery and child gender (*n* = 1804) and child’s language, communication, and motor skills at 3 years of age (*n* = 39,839), the eligible study population was 45,389 mother-child pairs. For the neurodevelopmental outcomes at 5 years our study population was 17,310 mother child pairs, with no additional missing information on the communication and motor skills.

### Maternal cell phone use during pregnancy

The use of cell phones during early pregnancy was assessed by a questionnaire administered at 17th weeks of gestation. Pregnant women were asked to report their frequency of talking on the cell phone by choosing 1 of the 4 fixed frequency answers: “seldom/never”, “few times a week”, “daily” and “more than an hour daily”. In our analysis, women were categorized into 4 groups of cell phone use in early pregnancy according to their answer in this question as: “no use”, “low use”, “medium use”, and “high use”. Similar information on maternal cell phone use was collected at 30th week of pregnancy (*n* = 44,339).

### Child language, communication and motor skills at 3 and 5 years

Early language development of the children at 3 years was assessed by the Dale and Bishop Grammar rating, in which the mother was asked to rate her child’s typical sentence structure by choosing one of the six response categories [13, 14]. The list of options is an ordinal grammar rating with the highest rate indicating the most complex use of language. We assessed the risk of having lower sentence complexity, by grouping any ratings bellow six (≤5) and using the highest rating as the reference group. We used this cut-off to capture potentially late language development, basing the rationale on the publication by Dale P.S. et al., where 11% of the typical children and 46% of the early language delay children scored ≤5 [13]. As we do not expect children with severe language delay in our study population, we used this cut-off for capturing children with inflated language scores, i.e. children who score lower than the typical developing groups. Children categorized as “not yet talking” (lowest rating) were excluded from our analysis (*n* = 103).

Communication skills at 3 and 5 years, were assessed by the “Ages and Stages” questionnaire (ASQ) [15]. We defined children with low communication skills, as those with score < 40 on the ASQ total score (0 to 60) at 3 years and those with score ≤ 30 on the ASQ total score (0 to 60) at 5 years. At 3 years, 524 (1.2%) children, and at 5 years, 95 (0.6%) children were ranked as having low communication skills, and 42 children were ranked with low communication skills in both time points.

Motor skills at 3 years were also assessed by the “Ages and Stages” questionnaire (ASQ) [15], and motor skills at 5 years by the “Child Development Inventory” questionnaire (CDI) [16]. We defined low motor skills at 3 and 5 years as having a score in the lowest tertile calculated for the included study sample. Due to skewed distributions of the scores the lowest tertile did not include the exact 33% of the children, but 32% of the 3-year olds and 23% of the 5-year olds, which were categorized as having low motor skills. The score cut-off reflecting the lowest tertile was 30 (score range 0–40) for 3 year olds and 10 (score range 0–12) for 5 year old children. Approximately 2069 children were ranked as having low motor skills in both time points assessed. More detailed information on the validity and scoring of the used instruments are presented in supplementary material (Additional file 1: Tables S1, S2a, b, S3a, b).

### Other characteristics

Several maternal socio-demographic, lifestyle and pregnancy related characteristics were examined as potential confounders of the associations under study, including: maternal age (years), maternal education (≤12 years/13–16 years/≥17 years), parental income (both parents low income/either parent high income/both parents high income), parity (primiparous/multiparous), maternal occupation (public sector or military/private sector or self-employed/other), computer screen use during pregnancy (yes/no), marital status (living with partner/other), smoking prior to and during pregnancy (no/occasionally/daily), alcohol consumption prior to and during pregnancy (never or <1 time per month/1–3 times per month/≥1 time per week), use of folic acid supplements during pregnancy (yes/no), pre-pregnancy body mass index (BMI; <18.5, 18.5–24.9, 25–29.9, ≥30 kg/m^2^), type of delivery (c-section/normal) and the length of gestation (in weeks).

During the first years of life the interaction of the child with the mother/caregiver can affect the child’s psychosocial and cognitive development [17]. The amount of talk in the child’s environment, including talkative mothers, can promote vocabulary output and syntactic skills, trough high language input [18]. We hypothesized that women with an extrovert personality would talk more and report higher cell phone use than those with lower score, which can promote child’s communication skills. Maternal extrovert personality was assessed by the International Personality Item Pool (IPIP) Big-Five factor markers via a questionnaire administered at the 5 –years follow-up [19]. The scoring of the included 10 items (5 positive and 5 negative) resulted to a continuous score from 10 to 50, and women were categorized as “low” (score < −1SD), “average” (−1SD < score < +1SD) and “high” (score > +1SD) on extraversion, as suggested by Goldberg et al. [19].

Additionally, we used the year of delivery to assess time trends of cell phone use. Even though the recruitment of MoBa was finished in 2008, meaning that the cell phone questions were answered up to 2008, there are women delivering at 2009. Paternal use of cell phones for the 6 months before the pregnancy was assessed by a questionnaire administered around 15 weeks of pregnancy, but only 20,424 (45%) of the fathers provided information on mobile phone use, due to delayed administration of the fathers’ questionnaire. In addition, two different questionnaire versions were administered with different fixed answers of the questions assessing mobile phone use. We have included the question with the answers similar to those the mothers had to answer for comparability reasons, using the same labeling of the categories as for the mothers.

Based on a-priori assumptions, breastfeeding duration until 18 months (no breastfeeding/1–6 months/7–13 months/>13 months), child gender (boy/girl), maternal depression and/or anxiety before and/or during pregnancy (yes/no) were also assessed.

Characteristics that were univariately related with both the exposure and the outcome at 3 years, were included in our adjusted models as confounders.

### Statistical analysis

We described the distribution and assessed the differences of maternal socio-demographic, lifestyle and pregnancy related characteristics by no use or any use of cell phone in early pregnancy. Further, in a bar graph we described the distribution of cell phone use in early pregnancy, by year of delivery.

The crude and adjusted associations between maternal cell phone use in early pregnancy and language, communication and motor skills of the children were assessed by crude and multiple logistic regression models. Two different classifications of the exposure variables were used: i) a bivariate of no use vs. any use, and ii) a 4-level variable of no use, low use, medium use and high use of cell phone in early pregnancy. In addition, two adjusted models were formed. First, one model with variables that were identified as confounders in univariate analyses and second, another model with the maternal extrovert personality score added. We performed complete case analysis of 45,389 mother-child pairs with the neurodevelopmental outcomes at 3 years and of 17,310 mother-child pairs with the neurodevelopmental outcomes at 5 years.

Further adjustment for maternal self-reported anxiety and/or depression during pregnancy did not modified our results, hence it was not included in the final models.

#### Sensitivity analyses

The association with language skills at 3 years was examined as the risk of having lower sentence complexity and, in sensitivity analysis, as the risk of having any of the four specific categories of language skills. We conducted all the analysis after excluding non-users, with low cell phone users constituting the reference group. In addition, we performed a stratified analysis by child’s gender to examine any gender-specific susceptibility.

Several studies have shown an exponential increase of mobile phone users from the mid to late 1990s’ in the Nordic countries as well [20–22]. Around 2003, mobile operators in Europe deployed Universal Mobile Telecommunications System networks (3G), which were upgraded in 2006, leading to a 4400 fold increase in data transmission rates [23]. In addition, from the mid- to late 2000s’ smartphones became more popular, including the introduction of the iPhone. Hence, we have stratified our analyses by these periods to investigate possible time trends in cell phone use. In addition, this categorisation provided groups with similar numbers of mother-child pairs (37%, 32%, 31% of the study population in each period group respectively). Further sensitivity analyses included stratified analysis by year of birth (1999–2004, 2005–2006, 2007–2009) to study the potential effect of changes in cell phone use. Since the number of non-users decreased substantially during the period 2005–2009, the exposure variable used was a 3-categories variable with low cell phone users as the reference group, after excluding non-users.

In addition, the association between paternal mobile phone use during pregnancy and child neurodevelopment was investigated as a sensitivity analysis. Approximately 16% of the women included in our study have participated in the MoBa study with more than one pregnancy. Hence, as a sensitivity analysis, we performed logistic regression analyses by taking into account the clusters of siblings within their mothers. Our study population changed from the 3 year to the 5 year follow-up due to loss of participants in addition to delayed administration of the questionnaire (approximately 3 years after the cohort had reached 5 years). We investigated whether the exposure variable and the confounders included in the models had a different distribution in each sub-sample.

All analyses were performed using STATA 12.1 (Stata Corporation, College Station, Texas).

## Results

Ten percent (9.8%, *n* = 4428) of women reported no use of cell phones in early pregnancy, 39% were categorized as low phone users and 4.3% as high users. Cell phone users were more likely to be younger, deliver after 2005, primiparous, highly educated, with higher income, and employed in the private sector or self-employed, compared to non-users (Table 1). Prior to their pregnancy, cell phone users were more likely to be occasional or daily smokers and frequent alcohol consumers compared with non-users. Maternal high extrovert personality score was related to any cell phone use and high phone use as well (7% of non-users, 9% of low users, 14% of average users and 26% of high users had high extrovert score). The same factors were related to high use of cell phone compared with low use (data not shown). Cell phone use was not substantially changed when re-assessed later in pregnancy (week 30th), with 77% remaining non-users and 46% remaining high users. The agreement of cell phone use between the two time points was 76% (Cohen’s kappa = 0.61). Paternal use of cell phone during pregnancy tended to follow maternal use (Cohen’s kappa = 0.17, agreement = 46%). Approximately, 23% of women non-users, 35% of women low users, 68% of women medium users and 25% of women high users were in the same cell phone use category as their partner. We observed no difference by category of maternal cell phone use related to marital status, pre-pregnancy BMI, smoking, alcohol consumption and folic acid supplements use during pregnancy, type of delivery, preterm birth, child gender and breastfeeding duration (data not shown).

**Table 1:** Distribution of maternal, pregnancy and child characteristics by category of cell phone use in early pregnancy

	Cell phone use in early pregnancy (*n* = 45,389)			
	No use (*n* = 4428)		Any use (40,961)	
	*N*	%	*N*	%
Maternal characteristics				
Maternal age				
< 30 years	1433	32.4	18,011	44.0
30–34 years	2275	51.4	18,053	44.1
≥ 35 years	720	16.2	4897	11.9
Year of delivery				
1999–2004	3208	74.6	13,465	33.0
2005–2006	834	19.4	13,562	33.2
2007–2009	256	6.0	13,823	33.8
Parity				
Primiparous	1364	29.5	21,264	50.0
Multiparous	3255	70.5	21,240	50.0
Education				
≤ 12 years	1507	34.0	11,111	27.1
13–16 years	2026	45.8	18,177	44.4
≥ 17 years	895	20.2	11,673	28.5
Parental income				
Both parents low income	1698	39.4	10,196	25.4
Either parent high income	2049	47.5	16,714	41.6
Both parents high income	567	13.1	13,239	33.0
Missing	926 (2.0%)			
Maternal occupation				
Public sector/military	2372	53.7	19,292	47.2
Private sector/self-employed	1274	28.9	16,161	39.5
Other	767	17.4	5424	13.3
Missing	99 (0.2%)			
Smoking prior to pregnancy				
No	3537	79.9	30,366	74.1
Occasionally	282	6.4	4129	10.1
Daily	609	13.7	6466	15.8
Alcohol consumption prior to pregnancy				
Never/less than once per month	1998	49.7	13,789	35.0
1–3 times/month	1317	32.7	14,328	36.4
≥ 1 time/week	707	17.6	11,289	28.6
Missing	1961 (4.3%)			
Maternal extrovert personality score				
Low	140	37.3	2228	21.2
Average	210	56.0	6943	66.1
High	25	6.7	1339	12.7
Missing	34,504 (76.0%)			
Parental phone use				
Frequency of cell phone use in early pregnancy^a^				
Low use	0	0	17,690	43.2
Medium use	0	0	21,292	52.0
High use	0	0	1979	4.8
Maternal cell phone use in late pregnancy				
No use	3340	76.7	1885	4.7
Low use	987	22.7	17,524	43.8
Medium use	27	0.6	19,287	48.2
High use	1	0.02	1288	3.2
Missing	1050 (2.3%)			
Paternal cell phone use during pregnancy				
No use	620	22.7	621	3.5
Low use	1023	37.4	4404	24.9
Medium use	956	35.0	10,509	59.4
High use	133	4.9	2158	12.2
Missing	24,965 (55.0%)			

*p*-value <0.001 for chi-square test for all the shown comparisons

^a^Percentages correspond to within rows percentages, not columns

**Fig. 1:**
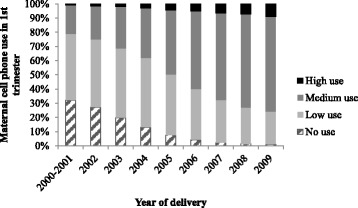
Maternal cell phone use in early pregnancy by year of delivery

The percentage of maternal high use of cell phone early in pregnancy increased by year of delivery, from 0.6% in 2000–2001 to 9.1% in 2009 (Fig. 1). The percentage of mothers with any cell phone use increased from 67.7% in 2000–2001 to 98.8% in 2009.

Approximatelly, 23% of the children were categorized as having lower sentence complexity at 3 years. Children born to cell phones users had 27% lower risk of having lower sentence complexity at 3 years of age, compared to children of non-users (Table 2). When the same association was assessed by none, low, medium and high maternal cell phone use a dose response association was found. After adjustement for confounders the observed association persisted; the risk of a child having lower sentence complexity at 3 years was 13%, 22% and 29% lower for low, medium and high maternal cell phone use, respectively, compared to children of non-users. After adding maternal extroversion score in the regression model, similar results were obtained.

**Table 2:** Associations between maternal cell phone use in early pregnancy and child’s lower sentence complexity at 3 years

	Risk for lower sentence complexity at 3 years		
	*N* (%) of cases	OR	95% CI
Maternal cell phone use in early pregnancy			
Crude model (*n* = 45,389)			
No use (*n* = 4428)	1232 (28%)	Ref.	
Any use (*n* = 40,961)	9028 (22%)	**0.73**	**0.68, 0.79**
Low use (*n* = 17,690)	4216 (24%)	**0.81**	**0.75, 0.87**
Medium use (*n* = 21,292)	4443 (21%)	**0.68**	**0.64, 0.74**
High use (*n* = 1979)	369 (19%)	**0.59**	**0.52, 0.68**
*p-for trend*		***<0.001***	
Adjusted model 1 (*n* = 45,389)			
No use (*n* = 4428)	1232 (28%)	Ref.	
Any use (*n* = 40,961)	9028 (22%)	**0.83**	**0.77, 0.89**
Low use (*n* = 17,690)	4216 (24%)	**0.87**	**0.81, 0.94**
Medium use (*n* = 21,292)	4443 (21%)	**0.78**	**0.72, 0.84**
High use (*n* = 1979)	369 (19%)	**0.71**	**0.62, 0.81**
*p-for trend*		***<0.001***	
Adjusted model 2 (n = 10,885)			
No use (*n* = 375)	111 (30%)	Ref.	
Any use (*n* = 10,510)	2155 (21%)	**0.73**	**0.58, 0.92**
Low use (*n* = 3688)	798 (22%)	**0.73**	**0.58, 0.93**
Medium use (*n* = 6220)	1241 (20%)	**0.72**	**0.57, 0.92**
High use (*n* = 602)	116 (19%)	0.75	0.55, 1.03
*p-for trend*		*0.173*	

*Bold fonts indicate statistical significant results, that are either confidence intervals that do not include 1 or p-for trend <0.05*

*N* (%) represents the number (and percentages) of children with lower sentence complexity (incomplete grammar; moderate language delay; severe language delay; speech problems) in each category of cell phone use

Adjusted model 1 includes parity, maternal age and education and year of delivery

Adjusted model 2 includes the variables of adjusted model 1 and maternal extrovert personality score (low/average/high)

After excluding non-users we observed a similar dose-response adjusted association for the risk of lower sentence complexity (medium cell phone users: OR = 0.90, 95% CI = 0.85, 0.95 and high cell phone users: OR = 0.82, 95% CI = 0.77, 1.02) (Additional file 2: Figure S1). Stratified analysis by gender provided similar results (Additional file 2: Figure S2A). Finally, when stratifying by year of delivery we found similar results as in the overall analysis, while with wider confidence intervals (Additional file 2: Figure S2B).

By examining the specified language skills as outcomes, we found a 14% (OR = 0.86, 95 CI% = 0.79,0.93) lower risk of having incomplete grammar and 31% (OR = 0.69, 95% CI = 0.59,0.81) lower risk of having moderate language delay at 3 years when the mother was a cell phone user compared to non-users (Additional file 1: Table S4). The associattion between cell phone use and moderate language delay was strengthen when adjusting for maternal extrovert score (OR = 0.49, 95% CI = 0.30,0.80). No association was observed between maternal cell phone use during pregnancy and the two most severe groups of language skills, severe language delay and speech problems (Additional file 1: Table S4).

Approximatelly, 1.2% of the 3 years-olds and 0.6% of the 5 years-olds included in this study, were categorized as having delayed communication skills. High maternal cell phone use during pregnancy was associated with 56% lower risk of delayed communication skills in 3-year old children, but adjustement for confounders removed this association (Table 3). We observed no association between maternal cell phone use during pregnancy and the risk for delayed communication skills in 5-year-old children. The null associations remained also after excluding non-users (Additional file 2: Figure S1).

**Table 3:** Associations between maternal cell phone use in early pregnancy and child’s low communication skills at 3 and 5 years

	Risk for low communication skills					
	At 3 years			At 5 years		
	N total/N with low skills (%)	OR	95% CI	N total/N with low skills (%)	OR	95% CI
Maternal cell phone use in early pregnancy						
Crude model						
No use	4428/60 (1.4%)	Ref.		1177/10 (0.9%)	Ref.	
Any use	40,961/464 (1.1%)	0.83	0.64, 1.09	16,133/85 (0.5%)	0.62	0.32, 1.19
Low use	17,690/215 (1.2%)	0.90	0.67, 1.19	6817/37 (0.5%)	0.64	0.32. 1.28
Medium use	21,292/237 (1.1%)	0.82	0.62, 1.09	8527/45 (0.5%)	0.62	0.31, 1.23
High use	1979/12 (0.6%)	**0.44**	**0.24, 0.83**	789/3 (0.4%)	0.45	0.12, 1.62
*p- for trend*		***0.018***			*0.234*	
Adjusted model 1						
No use	4428/60 (1.4%)	Ref.		1177/10 (0.9%)	Ref.	
Any use	40,961/464 (1.1%)	1.04	0.78, 1.38	16,133/85 (0.5%)	0.69	0.35, 1.36
Low use	17,690/215 (1.2%)	1.03	0.77, 1.39	6817/37 (0.5%)	0.69	0.34, 1.41
Medium use	21,292/237 (1.1%)	1.07	0.78, 1.45	8527/45 (0.5%)	0.70	0.34, 1.43
High use	1979/12 (0.6%)	0.62	0.33, 1.17	789/3 (0.4%)	0.56	0.15, 2.09
*p- for trend*		*0.724*			*0.438*	
Adjusted model 2						
No use	375/4 (1.1%)	Ref.		311/4 (1.3%)	Ref.	
Any use	10,510/86 (0.8%)	0.93	0.33, 2.59	8642/45 (0.5%)	0.65	0.22, 1.87
Low use	3688/29 (0.8%)	0.85	0.29, 2.45	3010/18 (0.6%)	0.65	0.21, 1.96
Medium use	6220/54 (0.9%)	1.02	0.36, 2.88	5131/26 (0.5%)	0.67	0.22, 2.00
High use	602/3 (0.5%)	0.65	0.14, 2.98	501/1 (0.2%)	0.33	0.04, 3.10
*p- for trend*		*0.847*			*0.506*	

*Bold fonts indicate statistical significant results, that are either confidence intervals that do not include 1 or p-for trend<0.05*

*N* (%) represent the number (and percentages) of children with low communication skills (score < 40 at 3 years and score ≤ 30 at 5 years) in each category of cell phone use. Total number of included mother-child pairs at 3 years are: *n* = 45,389 in crude and adjusted model 1 and *n* = 10,885 in adjusted model 2. Total number of included mother-child pairs at 5 years are: *n* = 17,310 in crude and adjusted model 1and *n* = 8953 in adjusted model 2

Adjusted model 1 includes parity, maternal age and education and year of delivery

Adjusted model 2 includes the variables of adjusted model 1 and maternal extrovert personality score in tertiles (low/average/high)

Regarding the associattions with motor skills, children of cell phone users had 18% lower risk of low motor skills (score in the lowest tertile). With regard to specific frequency of use the respective risks were 12%, 26% and 36% lower for low, medium and high cell phone users compared to non-users, in the adjusted model (Table 4). Ajustement for maternal extroversion score attenuated the estimates, while the dose-response trend persisted. After excluding non-users leaving the low-use as reference, the negative dose-response associattion remained (Additional file 2: Figure S1). Stratification by gender provided similar results (Additional file 2: Figure S3A). Similar results were obtained also after stratification by year of delivery (Additional file 2: Figure S3B). We observed no association between maternal cell phone use during pregnancy and the risk for motor skills score in the lowest tertile for 5-year-old children.

**Table 4:** Associations between maternal cell phone use in early pregnancy and child’s motor skills in the lowest tertile at 3 and 5 years

	Risk for motor skills score in the lowest tertile					
	At 3 years			At 5 years		
	N total/N with low skills (%)	OR	95% CI	N total/N with low skills (%)	OR	95% CI
Maternal cell phone use in early pregnancy						
Crude model						
No use	4428/1541 (34.8%)	Ref.		1177/241 (20.5%)	Ref.	
Any use	40,961/13,193 (32.2%)	**0.89**	**0.83, 0.95**	16,133/3685(22.8%)	1.15	0.99, 1.33
Low use	17,690/5945 (33.6%)	0.95	0.88, 1.02	6817/1562 (22.9%)	1.15	0.99, 1.34
Medium use	21,292/6669 (31.3%)	**0.85**	**0.80, 0.91**	8527/1936 (22.7%)	1.14	0.98, 1.33
High use	1979/579 (29.3%)	**0.77**	**0.69, 0.87**	789/187 (23.7%)	1.21	0.97, 1.50
*p- for trend*		***<0.001***			*0.247*	
Adjusted model 1						
No use	4428/1541 (34.8%)	Ref.		1177/241 (20.5%)	Ref.	
Any use	40,961/13,193 (32.2%)	**0.82**	**0.76, 0.87**	16,133/3685 (22.8%)	1.02	0.88, 1.19
Low use	17,690/5945 (33.6%)	**0.88**	**0.82, 0.94**	6817/1562 (22.9%)	1.07	0.91, 1.24
Medium use	21,292/6669 (31.3%)	**0.74**	**0.69, 0.80**	8527/1936 (22.7%)	0.98	0.83, 1.14
High use	1979/579 (29.3%)	**0.64**	**0.57, 0.72**	789/187 (23.7%)	1.00	0.81, 1.25
*p- for trend*		***<0.001***			*0.152*	
Adjusted model 2						
No use	375/132 (35.2%)	Ref.		311/74 (23.8%)	Ref.	
Any use	10,510/3544 (33.7%)	0.93	0.75, 1.16	8642/2123 (24.6%)	1.11	0.85, 1.46
Low use	3688/1323 (35.9%)	1.01	0.81, 1.27	3010/783 (26.0%)	1.17	0.89, 1.55
Medium use	6220/2040 (32.8%)	0.89	0.71, 1.11	5131/1214 (23.4%)	1.06	0.81, 1.40
High use	602/181 (30.1%)	0.79	0.60, 1.05	501/126 (24.2%)	1.20	0.85, 1.67
*p- for trend*		***0.001***			*0.552*	

*Bold fonts indicate statistical significant results, that are either confidence intervals that do not include 1 or p-for trend<0.05*

*N* (%) represent the number (and percentages) of children with low motor skills (score in the lowest tertile at 3 years and score in the lowest tertile at 5 years) in each category of cell phone use. Total number of included mother-child pairs at 3 years are: *n* = 45,389 in crude and adjusted model 1and *n* = 10,885 in adjusted model 2. Total number of included mother-child pairs at 5 years are: *n* = 17,310 in crude and adjusted model 1and *n* = 8953 in adjusted model 2

Adjusted model 1 includes parity, maternal age and education and year of delivery

Adjusted model 2 includes the variables of adjusted model 1 and maternal extrovert personality score in tertiles (low/average/high)

No substantial difference of the distribution of the exposure variable and the confounders was found between the study samples at the 3 and 5 years follow-up (Additional file 1: Table S6). To further investigate changes in effect estimates due to number of mother-child pairs in the model we performed sensitivity analysis of adjusted model 1 with the population of adjusted model 2 (*n* = 17,130 instead of *n* = 45,389), and the results were similar (Additional file 1: Table S6).

In addition, we run the same adjusted analyses as presented in models 1 in the tables after taking into account that some children are siblings and they cluster within the mother (Additional file 1: Table S7). All our estimates remained unchanged.

Finally, the associattion between paternal cell phone use and child’s neurodevelopmental outcomes at 3 and 5 years were in the same direction as observed for the maternal cell phone use, but weaker (Additional file 1: Table S8).

## Discussion

Overall, we observed a lower risk of reduced sentence complexity and low motor skills of 3 year-old children being associated with maternal cell phone use in early pregnancy compared with non-users. These associations were confirmed by dose-response trends, even after excluding non-users. Our unique findings do not support the hypothesis of adverse effects on children’s language, communication and motor skills due to maternal cell phone use during gestation.

The previous epidemiological studies investigating the effect of early exposure to cell phone and children cognitive and motor development in mother-child cohorts report no associations [7, 9]. In these studies, the outcomes were studied in infants and toddlers (≤3 y ears), while our study also includes pre-school children (5 years). Nevertheless, in the Spanish study cell phone users had children who scored higher in the Bayley mental scale and in the Danish study there was a trend for lower risk for cognitive/language delay at 6 months (Odds Ratio (OR) 0.9, 95% Confidence Intervals (CI) 0.7, 1.0) and for motor delay at 18 months (OR 0.9, 95% CI 0.8, 1.0), associated with maternal cell phone use. However discrepancies and similarities between studies can be related to the characteristics of the included population. The prevalence of non-users during pregnancy (6%) in our study, was similar to Vrijheid et al. (11%) (Spanish study) and Guxens et al. (6%) (Dutch study), with recruitments between 2003 and 2006, but lower than in Divan et al. (60%) (Danish study), with recruitment from 1997 to 2002 [7–9]. The characteristics related to maternal use of cell phone during pregnancy were similar to ours; younger women [6–9], highly educated, of higher socio-economic status [8, 9] and smokers and alcohol consumers before pregnancy [8]. However, in the studies by Divan et al., with adverse effects on child’s behavior, cell phone use was related to lower socio-economic status and higher maternal stress [5–7].

By investigating the specific groups in the Bishop-Dale tool, we observed lower risks of having incomplete grammar (i.e. “Children producing fairly complete sentences with incomplete grammar”) and moderate language delay (i.e. “Children producing short sentences”) in children of cell phone users. The latter observed association was higher (lower OR), but weaker (wider confidence intervals) when we adjusted for maternal extrovert personality score. We could assume that women could be divided into “heavy talkers” and “moderate talkers”, regardless of extraversion, and those women who talk a lot using their cell phones might also talk a lot to their child, explaining the association between heavy phone use and lack of reduced sentence complexity in the children. Hence, enhanced maternal-child communication among cell phone users could explain our findings.

We found no association with severe delay in expressive language or speech problems, as assessed by the Bishop-Dale tool, or severe delay in expressive/receptive language as assessed by the ASQ. In a recent case-control study of 77 cases of 3–5 year old children with clinically diagnosed speech problems and 35 controls, a higher prenatal exposure to cell phone was reported for the cases, while it was not clear whether the authors adjusted for potential confounders [24].

The neurobiological substrate in fine and gross motor development involves various parts of the brain including the cerebral cortex, basal ganglia and the cerebellum. Language processing functions are regulated through multiple maturational mechanisms, but are increasingly influenced by higher level cortical control. There are several studies with animals exposed prenatally to various regimes of mobile phone radiation. The exposure depth of radiofrequency radiation is qualitatively different in animals and humans, since the penetration depth is limited and frequency dependent, implying that the human brain will be structurally differently exposed compared to a small animal (rat) brain, even at the same power and distance to the source. Hence, fetal exposure to maternal mobile phone use, and rats exposed to a mobile phone during pregnancy, are not comparable. Different experimental studies of rats exposed during gestation to EMF between 900 and 1800 MHz, via cell phones, provided no evidence of exposure-related change in the offspring’s hippocampus [25] and cerebellum [26] or cognitive deficits [27] and effects on learning skills and behavior [28]. On the other hand, prenatal and postnatal exposure to cell phone produced EMF was related to changes in the numbers of neurotransmitters [29, 30]. The concern for the human health effects is mainly driven by the evidence from animal studies but animal evidence is still inconsistent and the relevance of these findings for humans is uncertain. To add to the complexity of the issue, the estimation of fetus RF exposure induced by wireless communication systems is highly complex because the exposure depends on many parameters (source, usage, frequency, posture, age of fetus). Specific absorption models showed that the fetus is exposed to various RF-EMF from the mother holding her cell phone close to her head or her body, depending on the position of the fetus and the pregnancy week [31].

The bias of unmeasured confounding is a possibility in our study. Even though we have attempted to adjust for all potential confounders by including important socio-demographic characteristics as well as maternal personality and psychological factors, unmeasured confounding might have affected our findings, i.e. genetic or other lifestyle factors. We have also performed several sensitivity analyses in an attempt to evaluate associations in different population strata. Hence, other unmeasured factors might be related to a protective effect on the development of sentence complexity and motor skills, rather than the prenatal cell phone use in itself. Nevertheless, our findings make it less likely that exposure to EMF from cell phone use during pregnancy should be associated with adverse effects on neurodevelopment. In addition, the observed associations between paternal cell phone use and the studied outcomes in the child were in the same direction as the maternal associations, suggesting that the association may be caused by social background in general rather than by direct influence of mobile phone use in pregnancy, given that the agreement between maternal and paternal cell phone use was poor.

The large sample size is the main strength of our study and together with the Danish study [7] these are the largest studies investigating the association between maternal cell phone use and neurodevelopmental effects in children. The adjustment for maternal extrovert personality is a strength of our analysis, as well as the information on maternal anxiety and/or depression assessment during pregnancy. Finally, the span of the recruitment years can be considered as a strength of this study as we have had the opportunity to study the associations of maternal cell phone use in periods of technological advance and increasing cell phone use. On the other hand, changes on cell phone use over time can be considered a limitation as well, as they can introduce bias in our analysis. Nevertheless, when restricting to children born earlier (1999–2004), between 2005 and 2006 and later (2007–2009) when cell phone use may have been more homogenous, similar results were found.

A main limitation of our study is the self-reported cell phone use. The research group of the COSMOS study, a large international prospective cohort study examining the possible health effects of long-term cell phone use, found 59% perfect agreement between reported vs. traffic data of call frequency [32]. Hence, our analyses could be affected by misclassification bias. The misclassification of the exposure due to errors in self-reported cell phone use would be non-differential (ie, the same degree of misclassification to both mothers of children with and without low language, communication, motor skills), because this was a cohort study and use of cell phones was assessed long before the neurodevelopmental assessment of the child. Non-differential bias leads to attenuation of observed associations, meaning that without such misclassification the observed associations could have been stronger [33]. In addition, we did not have information of cordless phone use. The parental assessment of language development, communication and motor skills of the children might also have introduced a misclassification bias. However, in recent studies in the MoBa, it was noted that delay in language development as assessed by mother-filled questionnaires may be a sensitive outcome of neurodevelopment following even low grade exposure to known environmental neurotoxicants during pregnancy [34, 35]. Finally, we did not investigate possible health effects of the early age exposure to EMF-RF from the offspring’s use cell phones.

## Conclusions

For the first time we reported a beneficial association between maternal cell phone use during pregnancy and the child’s neurodevelopment, within a large prospective cohort study. Limitations common in observational studies, including unmeasured confounding are a probable explanation of this association. Nevertheless, our findings provide evidence that exposure to EMF-RF from cell phone during pregnancy is not associated with adverse neurodevelopment in the offspring at 3 or 5 years.

## Additional files

Additional file 1: Table S1. Language skills by the Bishop & Dale grammar rating at 3 years. **Table S2a.** Ages and Stages Questionnaires (ASQ) at 3 years for communication skills. **Table S2b.** Ages and Stages Questionnaires (ASQ) at 5 years for communication skills. **Table S3a.** Ages and Stages Questionnaires (ASQ) at 3 years for motor skills. **Table S3b.** Child Development Inventory (CDI) at 5 years for motor skills. **Table S4.** Associations between maternal cell phone use during early pregnancy and the Bishop-Dale rating measure of language skills at 3 years. **Table S5.** Distribution of exposure and main confounder variables by population sub-samples. **Table S6.** Association between maternal cell phone use during pregnancy and child’s neurodevelopmental outcomes at 3 years (*n* = 10,885 mother-child pairs) and 5 years (*n* = 8953 mother-child pairs) in the same study sample as in the adjusted model 2. **Table S7.** Association between maternal cell phone use in early pregnancy and child’s neurodevelopmental outcome sat 3 and 5 years, in analysis taking into account the cluster of siblings within the mother. **Table S8.** Association between paternal cell phone use and child’s neurodevelopmental outcomes at 3 and 5 years. (DOCX 30 kb)

Additional file 2: Figure S1. Adjusted association between maternal cell phone use in early pregnancy and the risk for lower sentence complexity at 3 years, low communication skills at 3 years and low communication skills at 5 years, after excluding non-users, low motor skills at 3 years and low motor skills at 5 years, after excluding non-users. **Figure S2.** Stratified analysis of the adjusted association between maternal cell phone use in early pregnancy and lower sentence complexity at 3 years, by A) gender and by B) year. **Figure S3.** Stratified analysis of the adjusted association between maternal cell phone use in early pregnancy and low motor skills at 3 years, by A) gender and B) year of delivery. (DOCX 3340 kb)

## References

[1] Alexander J, Brunborg G, Feychting M, Forsberg EM, Gismervik S, Haanes JV, Hamnerius Y, Hannevik M, Heimdal PE, Hillert L et al.: Low-level radiofrequency electromagnetic fields – an assessment of health risks and evaluation of regulatory practice. In*.*, vol. 2012:3. Oslo, Norway: Norwegian Institute of Public Health; 2012.

[2] Feychting M (2011). Mobile phones, radiofrequency fields, and health effects in children--epidemiological studies. Prog Biophys Mol Biol.

[3] Hardell L, Sage C (2008). Biological effects from electromagnetic field exposure and public exposure standards. Biomed Pharmacother.

[4] Otto M, von Muhlendahl KE (2007). Electromagnetic fields (EMF): do they play a role in children's environmental health (CEH)?. Int J Hyg Environ Health.

[5] Divan HA, Kheifets L, Obel C, Olsen J (2008). Prenatal and postnatal exposure to cell phone use and behavioral problems in children. Epidemiology.

[6] Divan HA, Kheifets L, Obel C, Olsen J (2012). Cell phone use and behavioural problems in young children. J Epidemiol Community Health.

[7] Divan HA, Kheifets L, Olsen J (2011). Prenatal cell phone use and developmental milestone delays among infants. Scand J Work Environ Health.

[8] Guxens M, van Eijsden M, Vermeulen R, Loomans E, Vrijkotte TG, Komhout H, van Strien RT, Huss A (2013). Maternal cell phone and cordless phone use during pregnancy and behaviour problems in 5-year-old children. J Epidemiol Community Health.

[9] Vrijheid M, Martinez D, Forns J, Guxens M, Julvez J, Ferrer M, Sunyer J (2010). Prenatal exposure to cell phone use and neurodevelopment at 14 months. Epidemiology.

[10] Poorman E, Gazmararian J, Parker RM, Yang B, Elon L (2015). Use of text messaging for maternal and infant health: a systematic review of the literature. Matern Child Health J.

[11] Vodopivec-Jamsek V, de Jongh T, Gurol-Urganci I, Atun R, Car J (2012). Mobile phone messaging for preventive health care. Cochrane Database Syst Rev.

[12] Magnus P, Birke C, Vejrup K, Haugan A, Alsaker E, Daltveit AK, Handal M, Haugen M, Høiseth G, Knudsen GP, et al. Cohort profile update: the Norwegian mother and child cohort study (MoBa). Int J Epidemiol. 2016; 10.1093/ije/dyw029 27063603

[13] Dale PS, Price TS, Bishop DV, Plomin R (2003). Outcomes of early language delay: I. Predicting persistent and transient language difficulties at 3 and 4 years. Journal of speech, language, and hearing research : JSLHR.

[14] Bishop DV, Price TS, Dale PS, Plomin R (2003). Outcomes of early language delay: II. Etiology of transient and persistent language difficulties. Journal of speech, language, and hearing research : JSLHR.

[15] Squires J, Potter L, Bricker D (1999). The ASQ User's guide.

[16] Ireton H, Glascoe FP (1995). Assessing children's development using parents' reports. The child development inventory. Clin Pediatr.

[17] Kendrick D, Elkan R, Hewitt M, Dewey M, Blair M, Robinson J, Williams D, Brummell K (2000). Does home visiting improve parenting and the quality of the home environment? A systematic review and meta analysis. Arch Dis Child.

[18] Huttenlocher J (1998). Language input and language growth. Prev Med.

[19] Goldberg LR: A broad-bandwidth, public-domain, personality inventory measuring the lower-level facets of several five-factor models. In: Personality psychology in Europe Volume 7, edn. Edited by Mervielde I, Deary IJ, Fruyt FD, Ostendorf F. Tilburg, The Netherlands: Tilburg University Press; 1999: 7–28.

[20] Swerdlow AJ, Feychting M, Green AC, Leeka Kheifets LK, Savitz DA (2011). International Commission for non-Ionizing Radiation Protection Standing Committee on E: mobile phones, brain tumors, and the interphone study: where are we now?. Environ Health Perspect.

[21] Little MP, Rajaraman P, Curtis RE, Devesa SS, Inskip PD, Check DP, Linet MS (2012). Mobile phone use and glioma risk: comparison of epidemiological study results with incidence trends in the United States. BMJ.

[22] Deltour I, Johansen C, Auvinen A, Feychting M, Klaeboe L, Schuz J (2009). Time trends in brain tumor incidence rates in Denmark, Finland, Norway, and Sweden, 1974-2003. J Natl Cancer Inst.

[23] Whitehead M, Philips T, Page M, Molina M: European Mobile Industry Observatory. In*.*; 2011.

[24] Zarei S, Mortazavi SM, Mehdizadeh AR, Jalalipour M, Borzou S, Taeb S, Haghani M, Mortazavi SA, Shojaei-Fard MB, Nematollahi S (2015). A challenging issue in the etiology of speech problems: the effect of maternal exposure to electromagnetic fields on speech problems in the offspring. J Biomed Phys Eng.

[25] Ragbetli MC, Aydinlioglu A, Koyun N, Ragbetli C, Karayel M (2009). Effect of prenatal exposure to mobile phone on pyramidal cell numbers in the mouse hippocampus: a stereological study. Int J Neurosci.

[26] Haghani M, Shabani M, Moazzami K (2013). Maternal mobile phone exposure adversely affects the electrophysiological properties of Purkinje neurons in rat offspring. Neuroscience.

[27] Bornhausen M, Scheingraber H (2000). Prenatal exposure to 900 MHz, cell-phone electromagnetic fields had no effect on operant-behavior performances of adult rats. Bioelectromagnetics.

[28] Klose M, Grote K, Spathmann O, Streckert J, Clemens M, Hansen VW, Lerchl A (2014). Effects of early-onset radiofrequency electromagnetic field exposure (GSM 900 MHz) on behavior and memory in rats. Radiat Res.

[29] Celikozlu SD, Ozyurt MS, Cimbiz A, Yardimoglu MY, Cayci MK, Ozay Y (2012). The effects of long-term exposure of magnetic field via 900-MHz GSM radiation on some biochemical parameters and brain histology in rats. Electromagn Biol Med.

[30] Jing J, Yuhua Z, Xiao-qian Y, Rongping J, Dong-mei G, Xi C (2012). The influence of microwave radiation from cellular phone on fetal rat brain. Electromagn Biol Med.

[31] Varsier N, Dahdouh S, Serrurier A, De la Plata JP, Anquez J, Angelini ED, Bloch I, Wiart J (2014). Influence of pregnancy stage and fetus position on the whole-body and local exposure of the fetus to RF-EMF. Phys Med Biol.

[32] Hillert L, Fremling K, Smith RB, Heinavaara S, Ahlbom A, Elliott P, Auvinen A, Toledano MB. COSMOS Study: Validation of Self-Reported Mobile Phone Use. Epidemiology. 2012;23 (ISEE 2012 Conference Abstracts 5S).

[33] Blair A, Stewart P, Lubin JH, Forastiere F (2007). Methodological issues regarding confounding and exposure misclassification in epidemiological studies of occupational exposures. Am J Ind Med.

[34] Caspersen IH, Haugen M, Schjolberg S, Vejrup K, Knutsen HK, Brantsaeter AL, Meltzer HM, Alexander J, Magnus P, Kvalem HE (2016). Maternal dietary exposure to dioxins and polychlorinated biphenyls (PCBs) is associated with language delay in 3year old Norwegian children. Environ Int.

[35] Vejrup K, Schjolberg S, Knutsen HK, Kvalem HE, Brantsaeter AL, Meltzer HM, Alexander J, Magnus P, Haugen M (2016). Prenatal methylmercury exposure and language delay at three years of age in the Norwegian mother and child cohort study. Environ Int.

