# Supplementary material for: Maternal cell phone use in early pregnancy and child’s language, communication and motor skills at 3 and 5 years: the Norwegian mother and child cohort study (MoBa)
Source: BMC Public Health. 2017 Sep 5;17:685. doi: 10.1186/s12889-017-4672-2 (PMC5584361; doi:10.1186/s12889-017-4672-2)
Supplement: Supplementary file 1 — Language skills by the Bishop & Dale grammar rating at 3 years. Table S2a. Ages and Stages Questionnaires (ASQ) at 3 years for communication skills. Table S2b. Ages and Stages Questionnaires (ASQ) at 5 years for communication skills. Table S3a. Ages and Stages Questionnaires (ASQ) at 3 years for motor skills. Table S3b. Child Development Inventory (CDI) at 5 years for motor skills. Table S4. Associations between maternal cell phone use during early pregnancy and the Bishop-Dale rating measure of language skills at 3 years. Table S5. Distribution of exposure and main confounder variables by population sub-samples. Table S6. Association between maternal cell phone use during pregnancy and child’s neurodevelopmental outcomes at 3 years (n = 10,885 mother-child pairs) and 5 years (n = 8953 mother-child pairs) in the same study sample as in the adjusted model 2. Table S7. Association between maternal cell phone use in early pregnancy and child’s neurodevelopmental outcome sat 3 and 5 years, in analysis taking into account the cluster of siblings within the mother. Table S8. Association between paternal cell phone use and child’s neurodevelopmental outcomes at 3 and 5 years. (DOCX 30 kb) [file 12889_2017_4672_MOESM1_ESM.docx]

**Supplemental Tables**

# **Supplemental Table 1**. Language skills by the Bishop & Dale grammar rating at 3 years

|  | **Response categories in questionnaire** | **Description** | **Rating** |
| --- | --- | --- | --- |
| **1** | Not yet talking |  | Excluded (n=103) |
| **2** | He/she is talking, but you can’t understand him/her | Children having unintelligible speech | Speech problems |
| **3** | Talking in one-word utterances, such as “milk” or “down” | Children having minimal expressive language | Severe language delay |
| **4** | Talking in 2- to 3-word phrases, such as “me got ball” or “give doll” | Children producing short sentences | Moderate language delay |
| **5** | Talking in fairly complete sentences, such as “I got a doll” or “can I go outside?” | Children producing fairly complete sentences with incomplete grammar | Incomplete grammar |
| **6** | Talking in long and complicated sentences, such as “when I went to the park, I went on the swings” or “I saw a man standing on the corner” | Children producing long and complicated sentences with complete grammar | No language delay **(Reference category)** |

Footnotes: The Dale & Bishop rating tool is a measure of expressive language and sentence complexity. Language development is a common marker for neurodevelopment in toddlers ^1,2^. Additionally, parental reporting is generally a valid measure of child’s early expressive vocabulary, especially for assessing language delay ^3^. In-depth assessment of a subsample of 425 children in the MoBa study has been conducted to examine the validity of the measure; a high consistency was found between the in-depth assessment of language and speech development and maternal reports in the questionnaire^4^.

# **Supplemental Table** **2a.** Ages and Stages Questionnaires (ASQ) at 3 years for communication skills.

|  | **Understanding what others say and being able to communicate** | **Response options** |
| --- | --- | --- |
| 1 | Without showing him/her first, does your child point to the correct picture when you say,  “Where is the cat” or “Where is the dog”? Your child must only point at the correct picture | 1-Yes  2-Sometimes  3-Not yet |
| 2 | When you ask your child to point to his/her eyes, nose, hair, feet, ears, and so forth, does he/she correctly point to at least seven body parts? (The child can point to parts of himself/herself, you, or a doll.) |  |
| 3 | Does your child make sentences that are three or four words long? |  |
| 4 | Without giving him/her help by pointing or using gestures, ask your child to “Put the shoe on the table” and “Put the book under the chair”. Does your child carry out both of these directions correctly? |  |
| 5 | When looking at a picture book, does your child tell you what is happening or what action is taking place in the picture? (For example, “Barking”, “Running”, “Eating” and “Crying”?) You may ask, “What is the dog (or boy) doing?” |  |
| 6 | Can your child tell you at least two things about an object he/she is familiar with? If you say, for example, “Tell me about your ball”, will your child answer by saying something like “It is round, I can throw it, it is big”? |  |

# **Supplemental Table** **2b**. Ages and Stages Questionnaires (ASQ) at 5 years for communication skills.

|  | **The child’s ability to understand and tell** | **Response options** |
| --- | --- | --- |
| 1 | Without giving your child help by pointing or repeating directions, does your child follow three directions that are *unrelated* to one another? Give all three directions before your child starts. For example, you may ask your child to “Clap your hands, walk to the door, and sit down” or “Give me the pen, open the book, and stand up.” | 1-Yes  2-Sometimes  3-Not yet |
| 2 | Does your child use four- and five- word sentences? For example, does your child say, “I want the car”? |  |
| 3 | When talking about something that already happened, does your child use words that end in “ed” such as *walked, jumped* or *played*? Ask your child questions, such as “How did you get to the store?” (“We walked.”) “What did you do at your friend’s house?” (“We played.”) |  |
| 4 | Does your child use comparison words, such as *heavier, stronger* or *shorter*? Ask your child questions, such as “A car is *big*, but a bus is _____” (bigger); “A cat is *heavy*, but a man is ____” (heavier); A TV is *small*, but a book is ____ ” (smaller). |  |
| 5 | Does your child answer the following questions: 1. “What do you do when you are hungry?” (Acceptable answers include: “Get food”, “Eat”, “Ask for something to eat”, and “Have a snack”.) 2. “What do you do when you are tired?” (Acceptable answers include: “Take a nap”, “Rest”, “Go to sleep”, “Go to bed”, “Lie down”, and “Sit down.”) |  |
| 6 | Does your child repeat the sentences shown below back to you, without any mistakes? You may repeat each sentence one time. Mark “yes” if your child repeats both sentences without mistakes or “sometimes” if your child repeats one sentence without mistakes. “Jane hides her shoes for Maria to find.” “Al read the blue book under his bed.” |  |

Footnotes: The ASQ is a series of parent-completed screening questionnaires for child development, specific to ages from 4 to 60 months consisting of five scales: Communication, Gross Motor, Fine Motor, Problem Solving, and Personal-Social. ^5^. The Norwegian version of the ASQ has been validated previously ^6^.

Regarding the assessment of communication skills, the instrument used consists of six questions answered by the mother. In the 3 years questionnaire, four of these questions were from the 36 months scale, one item from the 18 months scale and one from the 48 months scale. In the 5 years questionnaire all six questions were from the 60 months scale. Each of the six items in the questionnaires had three response categories and was scored as follows: yes=10 points, sometimes=5 points and not yet=0 points, resulting in a continuous score from 0 to 60 ^5^**.**

# **Supplemental Table** **3a.** Ages and Stages Questionnaires (ASQ) at 3 years for motor skills.

|  | **About your child’s motor development** | **Response options** |
| --- | --- | --- |
| 1 | Can your child kick a ball by swinging his/her leg forward without holding onto anything for support? | 1-Yes  2-Sometimes  3-Not yet |
| 2 | Can your child catch a large ball with both hands? |  |
| 3 | When drawing, does your child hold a pencil, crayon or pen between his/her fingers  and thumb like an adult does? |  |
| 4 | Can your child undo one or more buttons? |  |

# **Supplemental Table** **3b.** Child Development Inventory (CDI) at 5 years for motor skills.

|  | **About motor skills** | **Response options** |
| --- | --- | --- |
| 1 | Do you think your child walks, runs, and climbs like other children at the same age? | 1-No  2-Yes |
| 2 | Able to stand on one foot for at least 5 sec without problems keeping balance |  |
| 3 | Hops, on one foot , many times, without support |  |
| 4 | Plays “catch” with other children; throwing to him/her and catching the ball at least half the time |  |
| 5 | Swings on a swing, pumping by self |  |
| 6 | Rides a two-wheeled bike, with or without training wheels |  |
| 7 | Puts together a puzzle with nine or more pieces |  |
| 8 | Draws or copies a square with straight corners |  |
| 9 | Cuts with scissors, following a simple outline or pattern |  |
| 10 | Draw pictures of complete people that have at least head: with eyes-nose-mouth; body. Arms and legs,  hands and feet (need to do all seven for a yes) |  |
| 11 | Colures within the lines in a coloring book |  |
| 12 | Does your child show interest in and likes to participate in sports or active games requiring good motor skills? |  |

Footnotes: For the assessment of motor skills, the applied instrument at 3 years consists of four questions and at 5 years of twelve questions, answered by the mother. For the 3 year olds, each of the four items in the questionnaires had three response categories and was scored as follows: yes=10 points, sometimes=5 points and not yet=0 points, resulting in a continuous score ranging from 0 to 40, with 84.2% of our sample scoring equal or above 30. For the 5 year olds, the scoring was 0 points if the answer was "no" and 1 point if the answer was "yes", resulting in a score from 0 to 12, with 57.5% of the included children scoring 12. Both measures include items relating to fine- and gross motor skills.

# **Supplemental Table 4.** Associations between maternal cell phone use during early pregnancy and the Bishop-Dale rating measure of language skills at 3 years.

|  | Bishop-Dale rating of language skills at 3 years | | | | | | | | | | | |
| --- | --- | --- | --- | --- | --- | --- | --- | --- | --- | --- | --- | --- |
|  | Incomplete grammar | | | Moderate language delay | | | Severe language delay | | | Speech problems | | |
|  | N | OR | 95%CI | N | OR | 95%CI | N | OR | 95%CI | N | OR | 95%CI |
| Maternal cell phone use in early pregnancy | | | | | |  |  |  |  |  |  |  |
| ***Crude model*** | | | | | | | | | | | | |
| No use | 995 | Ref. |  | 213 | Ref. |  | 7 | Ref. |  | 17 | Ref. |  |
| Any use | 7,567 | **0.76** | **0.71, 0.82** | 1,267 | **0.60** | **0.51, 0.69** | 52 | 0.74 | 0.34, 1.64 | 142 | 0.84 | 0.50, 1.38 |
| ***Adjusted model 1*** | | | | | | | | | | | | |
| No use | 995 | Ref. |  | 213 | Ref. |  | 7 | Ref. |  | 17 | Ref. |  |
| Any use | 7,567 | **0.86** | **0.79, 0.93** | 1,267 | **0.69** | **0.59, 0.81** | 52 | 1.16 | 0.51, 2.65 | 142 | 0.89 | 0.53, 1.47 |
| ***Adjusted model 2*** | | | | | | | | | | | | |
| No use | 89 | Ref. |  | 20 | Ref. |  | 0 | Ref. |  | 2 | Ref. |  |
| Any use | 1,861 | 0.78 | 0.61, 1.01 | 258 | **0.49** | **0.30, 0.80** | 6 | n.e. |  | 30 | 0.44 | 0.13, 1.50 |
| Adjusted model 1 includes parity, maternal age and education and year of delivery. | | | | | | | | | | | | |
| Adjusted model 2 includes the variables of adjusted model 1 and maternal extrovert personality score (low/average/high). | | | | | | | | | | | | |
| n.e.: not estimated | | | | | | | | | | | | |

# **Supplementary Table 5.** Distribution of exposure and main confounder variables by population sub-samples.

|  | Baseline population-  At birth^a^ (N=85,228) | Included population-  3 years follow-up (n=45,389) | Included population-  5 years follow-up (N=17,310) |
| --- | --- | --- | --- |
| Maternal cell phone use in early pregnancy |  |  |  |
| No | 8,515 (10.0%) | 4,428 (9.8%) | 1,177 (6.8%) |
| Low | 31,906 (37.4%) | 17,690 (39.0%) | 6,817 (39.4%) |
| Medium | 40,813 (47.9) | 21,292 (46.9%) | 8,527 (49.2%) |
| High | 3,994 (4.7) | 1,979 (4.3%) | 789 (4.6%) |
| Parity |  |  |  |
| Nulliparous | 37,738 (44.3%) | 21,865 (48.2%) | 8,386 (48.4%) |
| Multiparous | 47,490 (55.7%) | 23,524 (51.8%) | 8,924 (51.6%) |
| Maternal education |  |  |  |
| Low | 27,481 (32.2%) | 12,618 (27.8%) | 4,301 (24.9%) |
| Medium | 35,963 (42.2% | 20,203 (44.5%) | 7,950 (45.9%) |
| High | 21,784 (25.6%) | 12,568 (27.7%) | 5,059 (29.2%) |
| Year of delivery |  |  |  |
| 1999-2004 | 32,559 (38.2%) | 16,821 (37.1%) | 3,884 (22.4%) |
| 2005-2006 | 26,304 (30.9%) | 14,453 (31.8%) | 9,026 (52.1%) |
| 2007-2009 | 26,365 (30.9%) | 14,115 (31.1%) | 4,400 (25.4%) |
| Maternal age (mean, SD) | 30.1 (4.6) | 30.3 (4.4) | 30.5 (4.4) |
|  |  |  |  |
| Maternal extrovert personality score assessed at the 5 years follow-up ^b^ | (n=14,096) | (n=10,885) | (n=8,953) |
| Low | 3,040 (21.6%) | 2,368 (21.8%) | 1,940 (21.7%) |
| Average | 9,287 (65.9%) | 7,153 (65.7%) | 5,863 (65.5%) |
| High | 1,769 (12.5%) | 1,364 (12.5%) | 1,150 (12.8%) |
| ^a^ The baseline population includes participants with singleton, liveborn births with no congenital anomalies. | | | |
| ^b^ Maternal extrovert personality was assessed by questionnaires administered 5 years post-partum. Hence the number of women with available information does not correspond to the baseline population. | | | |

# **Supplementary Table 6.** Association between maternal cell phone use during pregnancy and child’s neurodevelopmental outcomes at 3 years (n=10,885 mother-child pairs) and 5 years (n=8,953 mother-child pairs) in the same study sample as in the adjusted model 2.

|  | Maternal cell phone use during pregnancy | | | | | | | |  |
| --- | --- | --- | --- | --- | --- | --- | --- | --- | --- |
|  | No use | | Low use | | Mediumuse | | High use | |  |
|  | OR | 95%CI | OR | 95%CI | OR | 95%CI | OR | 95%CI | *p-trend* |
| Risk for lower sentence complexity at 3 years | *1.00* |  | **0.77** | **0.60,0.98** | **0.71** | **0.55,0.91** | 0.74 | 0.54,1.03 | ***0.028*** |
| Risk for low communication skills | |  |  |  |  |  |  |  |  |
| At 3 years | *1.00* |  | 0.91 | 0.31, 2.61 | 0.87 | 0.30, 2.48 | 0.64 | 0.14, 2.93 | *0.603* |
| At 5 years | *1.00* |  | 0.52 | 0.17, 1.58 | 0.65 | 0.22, 1.92 | 0.25 | 0.03,2.33 | *0.659* |
| Risk for motor skills score in the lowest tertile | |  |  |  |  |  |  |  |  |
| At 3 years | *1.00* |  | 0.98 | 0.77, 1.25 | 0.83 | 0.66, 1.06 | **0.72** | **0.54, 0.98** | ***<0.001*** |
| At 5 years | *1.00* |  | 1.13 | 0.86, 1.49 | 1.02 | 0.78, 1.35 | 0.99 | 0.70, 1.40 | *0.166* |
| Models are adjusted for parity, maternal age and education and year of delivery. | | | | | | | | | |

# **Supplementary Table 7.** Association between maternal cell phone use in early pregnancy and child’s neurodevelopmental outcome sat 3 and 5 years, in analysis taking into account the cluster of siblings within the mother.

|  | No use | | Any use | | Low use | | Medium use | | High use | |  |
| --- | --- | --- | --- | --- | --- | --- | --- | --- | --- | --- | --- |
|  | OR | 95%CI | OR | 95%CI | OR | 95%CI | OR | 95%CI | OR | 95%CI | *p-trend* |
| Risk for lower sentence complexity at 3 years | *1.00* |  | **0.83** | **0.77,0.89** | **0.87** | **0.81,0.94** | **0.78** | **0.72,0.84** | **0.71** | **0.62,0.81** | *<0.001* |
| Risk for low communication skills | | |  |  |  |  |  |  |  |  |  |
| At 3 years | *1.00* |  | 1.04 | 0.77,1.39 | 1.03 | 0.76,1.40 | 1.07 | 0.77,1.48 | 0.62 | 0.33,1.18 | *0.728* |
| At 5 years | *1.00* |  | 0.69 | 0.35,1.39 | 0.69 | 0.34,1.43 | 0.70 | 0.33,1.46 | 0.56 | 0.15,2.15 | *0.464* |
| Risk for motor skills score in the lowest tertile | | |  |  |  |  |  |  |  |  |  |
| At 3 years | *1.00* |  | **0.82** | **0.76,0.88** | **0.88** | **0.82,0.94** | **0.74** | **0.69,0.80** | **0.64** | **0.57,0.72** | *<0.001* |
| At 5 years | *1.00* |  | 1.02 | 0.88,1.19 | 1.07 | 0.91,1.24 | 0.98 | 0.83,1.26 | 1.00 | 0.83,1.14 | *0.155* |
| Models are adjusted for parity, maternal age and education and year of delivery. | | | | | | | | | | | |

# **Supplementary Table 8.** Association between paternal cell phone use and child’s neurodevelopmental outcomes at 3 and 5 years.

|  | Paternal cell phone use during pregnancy | | | | | | | |  |
| --- | --- | --- | --- | --- | --- | --- | --- | --- | --- |
|  | No use | | Low use | | Medium use | | High use | |  |
|  | OR | 95%CI | OR | 95%CI | OR | 95%CI | OR | 95%CI | *p-trend* |
| Risk for lower sentence complexity at 3 years | *1.00* |  | 0.94 | 0.81,1.08 | 0.88 | 0.77,1.01 | 0.86 | 0.73,1.01 | ***0.020*** |
| Risk for low communication skills | |  |  |  |  |  |  |  |  |
| At 3 years | *1.00* |  | 0.80 | 0.48, 1.34 | 0.83 | 0.52, 1.36 | 0.96 | 0.54, 1.71 | *0.880* |
| At 5 years | *1.00* |  | 0.93 | 0.27, 3.25 | 0.53 | 0.16, 1.81 | 1.25 | 0.34,4.58 | *0.954* |
| Risk for motor skills score in the lowest tertile | |  |  |  |  |  |  |  |  |
| At 3 years | *1.00* |  | 0.92 | 0.81, 1.06 | **0.84** | **0.74, 0.95** | **0.77** | **0.66, 0.90** | ***<0.001*** |
| At 5 years | *1.00* |  | 0.92 | 0.71, 1.19 | 0.95 | 0.75, 1.22 | 1.00 | 0.76, 1.32 | *0.567* |
| Models are adjusted for parity, maternal age and education and year of delivery. | | | | | | | | | |

**References**

1. Colledge E, Bishop DV, Koeppen-Schomerus G, Price TS, Happe FG, Eley TC, Dale PS, Plomin R. The structure of language abilities at 4 years: a twin study. *Dev Psychol* 2002;**38**(5):749-57.

2. Viding E, Spinath FM, Price TS, Bishop DV, Dale PS, Plomin R. Genetic and environmental influence on language impairment in 4-year-old same-sex and opposite-sex twins. *J Child Psychol Psychiatry* 2004;**45**(2):315-25.

3. Ireton H, Glascoe FP. Assessing children's development using parents' reports. The Child Development Inventory. *Clin Pediatr (Phila)* 1995;**34**(5):248-55.

4. Roth C, Magnus P, Schjolberg S, Stoltenberg C, Suren P, McKeague IW, Davey Smith G, Reichborn-Kjennerud T, Susser E. Folic acid supplements in pregnancy and severe language delay in children. *JAMA* 2011;**306**(14):1566-73.

5. Squires J, Potter L, Bricker D. *The ASQ User's Guide* 2nd ed. Baltimore: Paul H. Brookes Publishing Co., 1999.

6. Richter J, Janson H. A validation study of the Norwegian version of the Ages and Stages Questionnaires. *Acta Paediatr* 2007;**96**(5):748-52.
