# Supplementary material for: Maternal cell phone use in early pregnancy and child’s language, communication and motor skills at 3 and 5 years: the Norwegian mother and child cohort study (MoBa)
Source: BMC Public Health. 2017 Sep 5;17:685. doi: 10.1186/s12889-017-4672-2 (PMC5584361; doi:10.1186/s12889-017-4672-2)
Supplement: Supplementary file 2 — Adjusted association between maternal cell phone use in early pregnancy and the risk for lower sentence complexity at 3 years, low communication skills at 3 years and low communication skills at 5 years, after excluding non-users, low motor skills at 3 years and low motor skills at 5 years, after excluding non-users. Figure S2. Stratified analysis of the adjusted association between maternal cell phone use in early pregnancy and lower sentence complexity at 3 years, by A) gender and by B) year. Figure S3. Stratified analysis of the adjusted association between maternal cell phone use in early pregnancy and low motor skills at 3 years, by A) gender and B) year of delivery. (DOCX 3340 kb) [file 12889_2017_4672_MOESM2_ESM.docx]

**Supplemental figures**

**Supplemental figure 1.** Adjusted association between maternal cell phone use in early pregnancy and the risk for lower sentence complexity at 3 years, low communication skills at 3 years and low communication skills at 5 years, after excluding non-users, low motor skills at 3 years and low motor skills at 5 years, after excluding non-users.

**
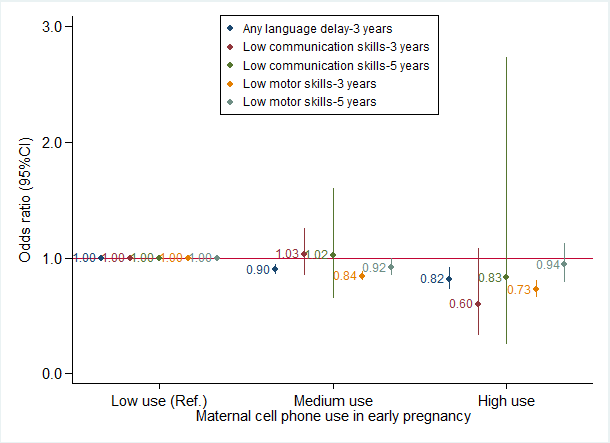
**

**Figure legend:** All models are adjusted for parity, maternal age and education and year of delivery**.**

**Supplemental Figure 2.** Stratified analysis of the adjusted association between maternal cell phone use in early pregnancy and lower sentence complexity at 3 years, by A) gender and by B) year.

**A)**


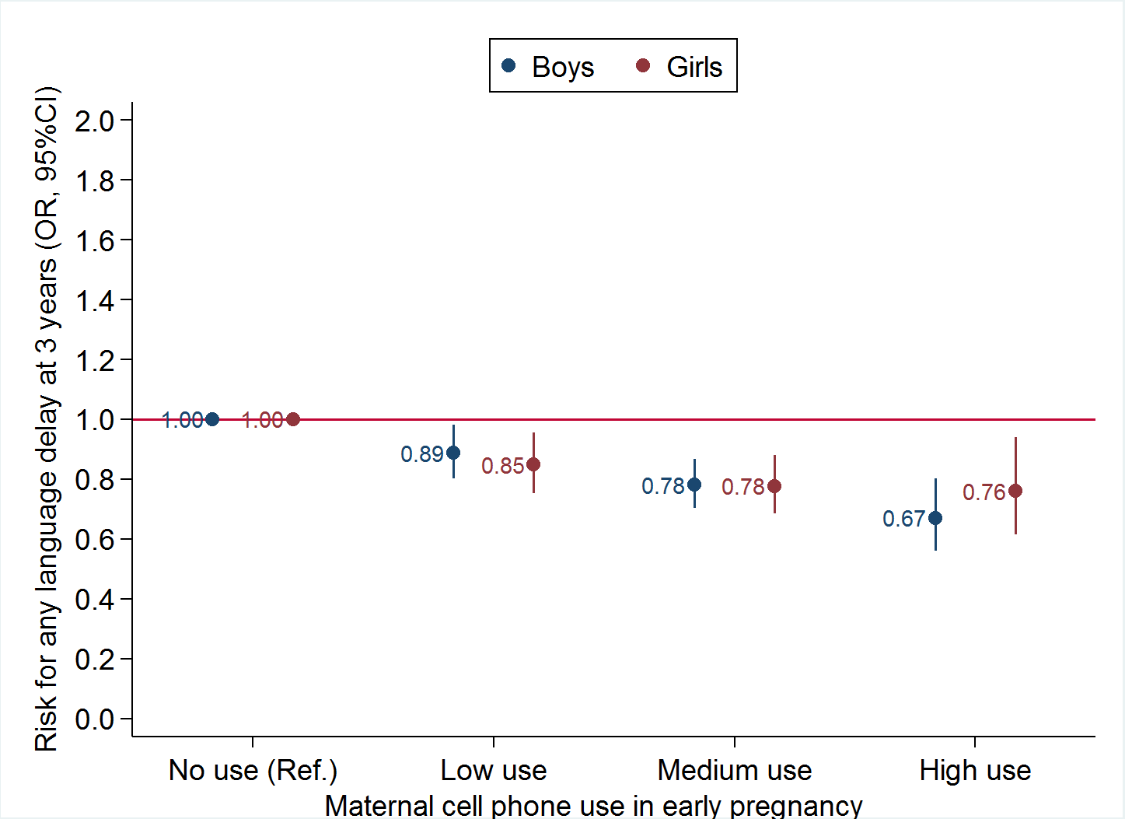


**B)**


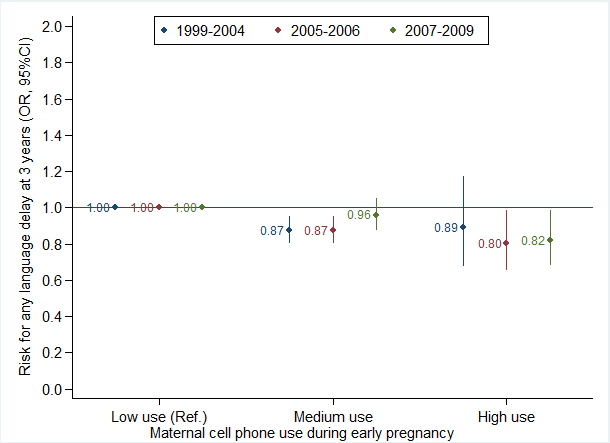


**Figure legend:** All models are adjusted for parity, maternal age and education and year of delivery**.**

**Supplemental Figure 3.** Stratified analysis of the adjusted association between maternal cell phone use in early pregnancy and low motor skills at 3 years, by A) gender and B) year of delivery.

**A)**

**
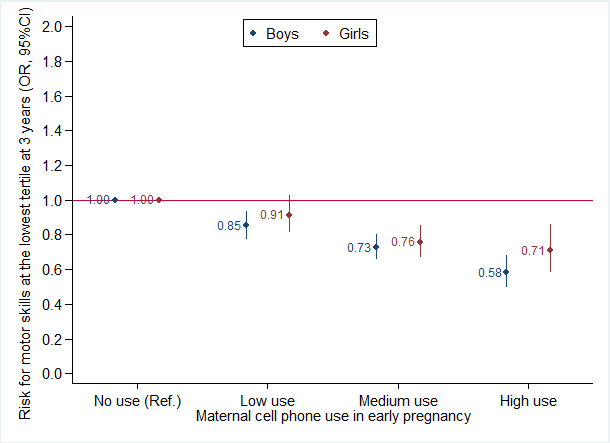
**

**B)**

**
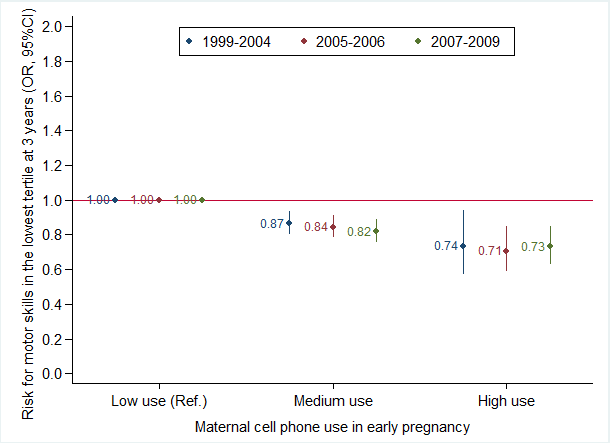
**

**Figure legend:** All models are adjusted for parity, maternal age and education and year of delivery**.**
